# Effects of a 6-Month Aerobic Exercise Intervention on Mood and Amygdala Functional Plasticity in Young Untrained Subjects

**DOI:** 10.3390/ijerph19106078

**Published:** 2022-05-17

**Authors:** Angelika Maurer, Julian Klein, Jannik Claus, Neeraj Upadhyay, Leonie Henschel, Jason Anthony Martin, Lukas Scheef, Marcel Daamen, Theresa Schörkmaier, Rüdiger Stirnberg, Tony Stöcker, Alexander Radbruch, Ulrike I. Attenberger, Martin Reuter, Henning Boecker

**Affiliations:** 1 Clinical Functional Imaging Lab, Department of Diagnostic and Interventional Radiology, University Hospital Bonn, Venusberg-Campus 1, 53127 Bonn, Germany; julian.klein@ukbonn.de (J.K.); jannik.claus@ukbonn.de (J.C.); neeraj.upadhyay@ukbonn.de (N.U.); jason.martin@ucc.ie (J.A.M.); scheef@hs-koblenz.de (L.S.); henning.boecker@ukbonn.de (H.B.); 2 German Center for Neurodegenerative Diseases, Venusberg-Campus 1, Building 99, 53127 Bonn, Germany; leonie.henschel@dzne.de (L.H.); marcel.daamen@dzne.de (M.D.); theresa.schoerkmaier@dzne.de (T.S.); ruediger.stirnberg@dzne.de (R.S.); tony.stoecker@dzne.de (T.S.); martin.reuter@dzne.de (M.R.); 3 Department of Neuroradiology, University Hospital Bonn, Venusberg-Campus 1, 53127 Bonn, Germany; alexander.radbruch@ukbonn.de; 4 Department of Diagnostic and Interventional Radiology, University Hospital Bonn, Venusberg-Campus 1, 53127 Bonn, Germany; ulrike.attenberger@ukbonn.de

**Keywords:** amygdala, functional connectivity, physical activity, exercise, mood, affect, fMRI, structural MRI, randomized controlled study, mental health

## Abstract

Acute exercise has beneficial effects on mood and is known to induce modulations in functional connectivity (FC) within the emotional network. However, the long-term effects of exercise on affective brain circuits remain largely unknown. Here, we investigated the effects of 6 months of regular exercise on mood, amygdala structure, and functional connectivity. This study comprised *N* = 18 healthy sedentary subjects assigned to an intervention group (IG; 23.9 ± 3.9 years; 3 trainings/week) and *N* = 10 subjects assigned to a passive control group (CG; 23.7 ± 4.2 years). At baseline and every two months, performance diagnostics, mood questionnaires, and structural and resting-state-fMRI were conducted. Amygdala-nuclei segmentation and amygdala-to-whole-brain FC analysis were performed. Linear mixed effects models and correlation analyses were conducted between FC, relVO_2max_, and mood scores. Data showed increases in relVO_2max_ exclusively in the IG. Stronger anticorrelation in amygdala-precuneus FC was found, along with a stronger positive correlation in the amygdala-temporal pole FC in the IG after 4 and 6 months, while mood and amygdala volume did not reveal significant interactions. The relVO_2max_/amygdala-temporal pole FC correlated positively, and the amygdala-precuneus/amygdala-temporal pole FC correlated negatively. Findings suggest that exercise induced long-term modulations of the amygdala FC with the precuneus and temporal pole, shedding light on potential mechanisms by which exercise has positive influences on mood-related networks, typically altered in affective disorders.

## 1. Introduction

The amygdala is a central component of the limbic system, playing a crucial role in various aspects of emotional behavior. Converging evidence has been gathered in functional neuroimaging studies over the last few decades to support its role as an integral hub region in neuronal networks serving emotion processing. There is meta-analytical evidence for physiological amygdala involvement in a broad range of emotional processes [1], while amygdala malfunctioning and remote neural circuit dysfunction have been identified as biological disease signatures of mood disorders, e.g. depression [2]. In depressive disorders, decreases in amygdala volume have been reported [3], particularly in unmedicated depressed patients [4]. Another characteristic feature at the functional level is amygdala overactivity [5], indicating hyperactive bottom-up processing in depressive disorders. Further, resting-state functional connectivity (rs-FC) of the amygdala is pathologically altered in adults with depression, suggesting hyperconnectivity between the amygdala and the hippocampus/parahippocampus and ventromedial orbitofrontal cortex (OFC), as well as hypoconnectivity between the amygdala and insula, inferior frontal gyrus (IFG), superior temporal gyrus, caudate, and cerebellum [6]. Adolescent patients with major depressive disorder (MDD) show a hyperconnectivity of the amygdala at rest with the precuneus and the IFG, while the amygdala is hypoconnected with the dorsolateral prefrontal cortex and the right IFG [6].

Recently, exercise has been proposed as an alternative non-pharmacological treatment in affective disorders [7]. Apart from its emerging role in patients, regular physical activity may also protect against the development of depression [8,9]. Given that psychosocial stress is increasing in our challenging and highly-demanding modern society, exercise may be effective in promoting general positive and mood stabilizing effects in healthy subjects.

To further explore the positive effects of long-term exercise on mood in sedentary but otherwise healthy populations, further research is needed. In particular, it seems highly relevant to investigate how regular exercise impacts on amygdala networks. Only recently, studies have begun to disentangle the mechanisms promoting affective changes induced by acute and long-term exercise in healthy subjects: Work from our group reported increases in rs-FC between the amygdala and anterior insula after acute high-intensity exercise, and FC changes correlated positively with the positive affect (PA) scale of the Positive and Negative Affect Schedule (PANAS) [10]. Another acute study, not specifically focusing on affective processing but investigating the effects of a single moderate-intensity exercise intervention on rs-FC, was able to show an increase in within-network FC of the affect and reward network (ARN), especially in the amygdala [11]. In a cross-sectional acute study by Ge et al. [12], subjects (males in late adolescence) underwent either a control condition (seated rest) or a single 30-min moderate-intensity exercise training. In each session, an rs-fMRI scan and the Profile of Mood State (POMS) was conducted. Subjects performing aerobic exercise showed increased FC between the right amygdala and the right OFC, and increased amygdala-OFC FC was associated with decreased depression scores. Regarding longitudinal exercise studies, there has been so far only one imaging study focusing on changes in mood (acquired using the POMS) induced by exercise and their relation to resting-state modifications in adults [13]. This study did not reveal any significant changes in the amygdala; however, an increase in rs-FC between the parahippocampal gyrus and the superior temporal gyrus/superior temporal pole was found, being significantly negatively correlated with total mood disturbances of the POMS, thus indicating that a higher FC in these brain areas accompany improvements in mood.

Due to the lack of longitudinal brain imaging studies investigating affective changes in healthy subjects, the present exploratory study aimed to investigate the effects of six months of extensive interval training on amygdala structure and function. Based on the results of the previous literature and the fact that many previous studies converge on the role of the amygdala, we expected changes in amygdala volume and changes in amygdala rs-FC to regions of the emotional network, in particular the insula, OFC, structures of the ARN, and the default mode network (DMN). Data were acquired every two months, allowing us to test when the first changes at the structural and functional levels occurred.

## 2. Material and Methods

### 2.1. Participants

In this study, sedentary men and women aged 18–35 years were recruited via flyer distribution at the local university and within social media. Prior to study inclusion, participants were interviewed regarding their physical activity level using a custom questionnaire acquiring frequency, time, and type of exercise within different life spans (up to 12 years of age, 13–18 years of age, after 18 years of age until inclusion). Subjects with prior histories as competitive athletes and/or regular physical exercise training in the last 2 years preceding this study were excluded. Further exclusion criteria were psychiatric, neurologic, or cardiovascular disease (current or in the past) and MRI contraindications such as pregnancy, claustrophobia, non-removable metal, and tattoos exceeding a certain size.

Participants were informed about the study, and written informed consent was obtained after detailed explanation of all tests, potential discomforts, risks, and procedures employed in the investigation. The study was approved by the Ethics Committee at the Medical Faculty of the Rheinische Friedrich-Wilhelms-Universität Bonn (no. 370/15), according to national legislation and the Declaration of Helsinki.

### 2.2. Experimental Procedure

‘RUNSTUD’ is a randomized 6-month exercise intervention study in young healthy sedentary subjects, which included the acquisition of performance diagnostics (PD), MRI (3T and 7T), neuropsychological tests, pain measurements, and blood sampling for epigenetics every 2 months (DRKS-ID: DRKS00021460). The current work focused solely on 3T MRI data and neuropsychological tests in the context of mood. Other examinations of this study were not the focus of the current work.

Demographic characteristics such as age and education level were acquired using a sociodemographic questionnaire, and a verbal intelligence level was estimated via a vocabulary test [14]. Handedness was assessed using the Edinburgh-Handedness-Inventory [15]. Psychiatric questionnaires such as the Mini International Neuropsychiatric Interview (M.I.N.I, German version 5.0.0; [16]), trait anxiety of the State-Trait-Anxiety Inventory (STAI; [17]), and the Beck Depression Inventory (BDI-I; [18]) were utilized to screen for psychiatric symptomatology. Furthermore, the Fagerström Test for Nicotine Dependence (FTND; [19]) and a questionnaire for substance consumption were employed.

**Figure 1:**
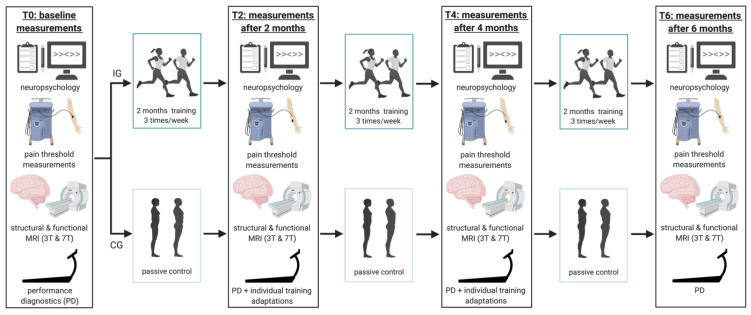
Study design. Figure created with Biorender.com.

Before the start of the study, a medical healthcare check involving an auscultation of the lung and heart, a 12-channel resting electrocardiogram, and an anamnestic questionnaire to exclude major physical health risks for subsequent exercise tests and trainings was performed. Then, subjects were randomly assigned to either the intervention group (IG) or the control group (CG). Due to an expected higher dropout rate for the IG, subjects were assigned in a 2:1 (intervention:control) ratio using the sequential list randomization method. At baseline (T0), all subjects underwent PD in form of a graded exercise test to determine individual fitness, a 3T MRI scan including structural and functional imaging, as well as questionnaires regarding affective behavior: PANAS [20], STAI state, BDI, and MoodMeter^®^ [21]. These measurements were repeated every two months throughout the intervention period (after two (T2), four (T4), and six months (T6)). The IG underwent a 6-month extensive interval training adjusted to individual fitness levels. The CG was instructed not to change their usual habits. An overview of the experimental procedure is presented in Figure 1.

### 2.3. Performance Diagnostics

The graded exercise test [22] was performed at T0, T2, T4, and T6 on a treadmill (PPS S70, Woodway GmBH, Weil am Rhein, Germany) with an initial speed of 6 km/h (incline set to 1%). Running speed was increased by 1 km/h every 3 min until volitional exhaustion. Oxygen uptake (VO_2_), metabolic respiratory exchange ratio (RER), and heart rate (HR) were recorded (Cortex meta-analyzer 3B, Leipzig, Germany, and Polar A360, Kempele, Finland). Capillary blood samples were collected from the fingertip during the last 15 s of each stage. The 20 µL capillary blood was mixed with 1 mL of hemolysis solution of the EBIO plus system and amperometric-enzymatically analyzed with EBIOplus (EKF Diagnostic, Magdeburg, Germany) to determine lactate concentration. At the same time, perceived exertion (RPE) was assessed using the 6-to-20 point Borg scale [23]. Voluntary exhaustion was defined as reaching at least two of the following criteria: level off in VO_2_, RER ≥ 1.10, high blood lactate levels (≥8 mmol/L), RPE of ≥18, and/or HR of ±10 bpm of the age-predicted maximum (220-age) [24]. In addition, relVO_2max_ was determined as the highest 30-s moving average of VO_2_ divided by body mass (mL/min/kg).

### 2.4. Intervention

During the intervention phase, subjects in the IG were required to complete 3 training sessions per week lasting 25–45 min. Of these, two running sessions per week were supervised on the treadmill in the laboratory environment, and one was performed at home in a flat terrain. Endurance training for the IG was designed as extensive interval training with 3- to 5-min intervals at 75–80% of HR_max_ and a 3- to 5-min active recovery with six to eight repetitions. Training intensity was individualized and adapted after each PD (2 and 4 months after baseline). Participants in the CG (passive control) were instructed to maintain their usual lifestyle (diet and exercise habits). Both groups were asked to wear a fitness tracker (Polar A360, Kempele, Finland) and to record any physical activity with the tracker. In the IG, the fitness tracker was additionally used to validate exercise performance.

### 2.5. Questionnaires

The BDI-I is a psychiatric self-report inventory with 21 items for the detection of depression and can be used to classify the respondent into the levels of no depression (0–10 points), mild to moderate level of depressive symptoms (11–17 points), ≥18 clinically relevant. The BDI was used for sample characterization only and to exclude depressive symptoms during the course of the study.

The STAI is an instrument for measuring anxiety and consists of two scales, each with 20 items. They are used to capture the intensity of current anxiety sensations (state-anxiety) and anxiety as a personality trait (the frequency of anxiety sensations in general; trait-anxiety). Items are rated on a 4-point Likert scale ranging from 1 “not at all” to 4 “very much”. Results can range from 20 “no anxiety” to 80 “maximum intensity of anxiety”. According to the original English version, clinically relevant anxiety scores are ≥39 [25].

The PANAS measures positive and negative affect (NA) with 10 items per subscale. A 5-point Likert scale ranging from 1 “not at all” to 5 “very much” is used.

The MoodMeter^®^ is a German questionnaire that is used to assess exercise-related mood changes [21]. A total of 32 adjectives on a 6-point Likert scale ranging from 0 “not at all” to 5 “completely” can be used to calculate 3 dimensions (perceived physical state (PEPS), psychological strain (PSYCHO), and motivational state (MOT)) with 4 sub-dimensions each (PEPS: physical energy, physical fitness, physical health, physical flexibility; PSYCHO: positive mood, calmness, recovery, relaxation; MOT: willingness to seek contact, social acceptance, readiness to strain, self-confidence).

### 2.6. MRI Acquisition

At each timepoint, subjects underwent a 3T MRI Scan at a SIEMENS MAGNETOM Skyra MRI (32 channel head-coil) located at the German Centre for Neurodegenerative Diseases in Bonn. Before each scan, participants were informed by a physician and screened for contraindications. Female participants were additionally tested for pregnancy. Within each scan, an inhouse-developed 3D echo-planar imaging sequence with 1 × 4_z2_ blipped-CAIPI sampling [26] was used to acquire rapid blood-oxygen-level-dependent fMRI timeseries with the following specifications: TR = 570 ms, TE = 30 ms, voxel size = 3 × 3 × 3 mm, field-of-view = 192 × 192 × 144 mm, water-selective excitation flip angle = 15° combined with spectral adiabatic fat inversion recovery, phase partial Fourier factor 7/8, semi-elliptical sampling [27], 48 slices per slab. Participants were asked to close their eyes and think of nothing in particular during the 10-min scan. Further, field mapping was performed with a double-echo spoiled gradient echo sequence (TR = 508 ms, TE = 4.92/7.38 ms, voxel size: 3 × 3 × 3 mm, flip angle 60°). Finally, anatomical T1w images were acquired using an inhouse-developed MP-RAGE sequence with 1 × 3_z1_ CAIPIRINHA and elliptical sampling [28] with the following specifications: sagittal slice orientation, voxel size = 1 × 1 × 1 mm, field-of-view = 192 × 192 × 144 mm, TR = 2.5 s, TI = 1.1 s, TE = 5 ms, flip angle = 7°, total scan duration: 2 min 53 s.

### 2.7. Amygdala Segmentation

T1w images were visually inspected for motion and acquisition artifacts and then automatically segmented using the longitudinal stream [29] in FreeSurfer 6.0 (http://surfer.nmr.mgh.harvard.edu/; accessed on 2 April 2022). Therein, an unbiased within-subject template space and image was created using robust, inverse consistent registration [30]. Common information from this within-subject template was then used to initialize several of the processing steps of the cross-sectional FreeSurfer pipeline (e.g., skull stripping, Talairach transformation, atlas registration, as well as spherical surface maps and parcellations), significantly increasing the reliability and statistical power of the longitudinal stream.

Amygdala nuclei segmentation was performed using the hippocampal subfields and amygdala nuclei module in FreeSurfer 7.0. Details on the procedure and amygdala nuclei segmentation can be found online (https://surfer.nmr.mgh.harvard.edu/fswiki/HippocampalSubfieldsAndNucleiOfAmygdala; accessed on 2 April 2022). Volumes were obtained of the whole left and right amygdala and 9 bilateral subfields, including seven nuclei (lateral nucleus, basal nucleus, accessory basal nucleus, central nucleus, medial nucleus, cortical nucleus, and paralaminal nucleus) and two transition areas (anterior amygdaloid area and corticoamygdaloid transition). Further, the estimated total intracranial volume (eTIV) was determined for each subject [31]. Afterwards, a visual quality control of the automatic amygdala segmentation was performed using the ENIGMA quality control protocol (http://enigma.ini.usc.edu/; accessed on 2 April 2022).

### 2.8. fMRI Data Preprocessing

Quality control of the raw MRI data was performed using MRIQC [32]. For the preprocessing, the standard pre-processing pipeline available in the fMRIPrep 20.0.6 toolbox [33], which is based on Nipype 1.4.2 [34], was used. 

#### 2.8.1. Anatomical Data Preprocessing

A total of 4 T1-weighted (T1w) images were found within the input BIDS dataset. All of them were corrected for intensity non-uniformity (INU) with N4BiasFieldCorrection [35], distributed with ANTs 2.2.0 ([36], RRID:SCR_004757). The T1w-reference was then skull-stripped with a Nipype [34] implementation of the antsBrainExtraction.sh workflow (from ANTs), using OASIS30ANTs as the target template. Brain tissue segmentation of cerebrospinal fluid (CSF), white-matter (WM), and gray-matter (GM) was performed on the brain-extracted T1w using fast (FSL 5.0.9, RRID:SCR_002823, [37]). A T1w-reference map was computed after registration of 4 T1w images (after INU-correction) using mri_robust_template (FreeSurfer 6.0.1, [30]). Brain surfaces were reconstructed using recon-all (FreeSurfer 6.0.1, RRID:SCR_001847, [38]), and the brain mask estimated previously was refined with a custom variation of the method to reconcile ANTs-derived and FreeSurfer-derived segmentations of the cortical gray-matter of Mindboggle (RRID:SCR_002438, [39]). Volume-based spatial normalization to standard space (MNI152NLin2009cAsym [40]) was performed through nonlinear registration with antsRegistration (ANTs 2.2.0), using brain-extracted versions of both the T1w reference and the T1w template. 

#### 2.8.2. Functional Data Preprocessing

For each of the rs-fMRI runs, the first seventeen rs-fMRI volumes were removed as dummy scans. For each of the 4 BOLD runs found per subject, the following preprocessing was performed. First, a reference volume and its skull-stripped version were generated using a custom methodology of fMRIPrep. A field map was estimated based on a phase-difference map calculated with a dual-echo GRE (gradient-recall echo) sequence, processed with a custom workflow of SDCFlows inspired by the epidewarp.fsl script and further improvements in HCP Pipelines [41]. The field map was then co-registered to the target EPI (echo-planar imaging) reference run and converted to a displacement field map (amenable to registration tools such as ANTs) with FSL’s fugue and other SDCflows tools. Based on the estimated susceptibility distortion, a corrected EPI reference was calculated for a more accurate co-registration with the anatomical reference. The BOLD reference was then co-registered to the T1w reference using bbregister (FreeSurfer), which implements boundary-based registration [42]. Co-registration was configured with six degrees of freedom. Head-motion parameters with respect to the BOLD reference (transformation matrices and six corresponding rotation and translation parameters) were estimated before any spatiotemporal filtering using mcflirt (FSL 5.0.9, [43]). The BOLD time-series were resampled onto their original, native space by applying a single, composite transform to correct for head-motion and susceptibility distortions. These resampled BOLD time-series will be referred to as preprocessed BOLD in the original space or just preprocessed BOLD. The BOLD time-series were resampled into standard space, generating a preprocessed BOLD run in MNI152NLin2009cAsym space. First, a reference volume and its skull-stripped version were generated using the custom methodology of fMRIPrep. Several confounding time-series were calculated based on the preprocessed BOLD: framewise displacement (FD), DVARS, and three region-wise global signals. FD and DVARS were calculated for each functional run, both using their implementations in Nipype (following the definitions by Power et al. [44]). The three global signals were extracted within the CSF, the WM, and the whole-brain masks. 

Additionally, a set of physiological regressors were extracted to allow for component-based noise correction (CompCor, [45]). Principal components were estimated after high-pass filtering the preprocessed BOLD time-series (using a discrete cosine filter with a 128 s cut-off) for the two CompCor variants: temporal (tCompCor) and anatomical (aCompCor). tCompCor components were then calculated from the top 5% variable voxels within a mask covering the subcortical regions. This subcortical mask was obtained by heavily eroding the brain mask, which ensures it does not include cortical GM regions. 

For aCompCor, components were calculated within the intersection of the aforementioned mask and the union of CSF and WM masks calculated in the T1w space, after their projection to the native space of each functional run (using the inverse BOLD-to-T1w transformation). For each CompCor decomposition, the k components with the largest singular values were retained, such that the retained components’ time-series were sufficient to explain 50 percent of variance across the nuisance mask (CSF, WM, combined, or temporal). The remaining components were dropped from consideration. The head-motion estimates calculated in the correction step were also placed within the corresponding confounds file. The confound time-series derived from the head motion estimates and global signals were expanded with the inclusion of temporal derivatives and quadratic terms for each [46]. Frames that exceeded a threshold of 0.5 mm FD or 1.5 standardized DVARS were annotated as motion outliers. In the denoising step, the aCompCor method, which performs well in removing cardiac and respiratory noise [47,48], was used. After smoothing the pre-processed data (5 mm FWHM), it was opted for removal of 10 aCompcor components, eight cosine function values, and 12 head motion parameters in a single step using the 3dTproject function from the AFNI toolbox [49,50].

The above boilerplate text was automatically generated by fMRIPrep with the express intention that users should copy and paste this text into their manuscripts unchanged. It is released under the CC0license.

#### 2.8.3. Seed-to-Whole-Brain Analysis

In order to perform a seed-to-whole-brain analysis of changes in the amygdala FC, right and left amygdala seed regions were defined using the Harvard-Oxford Subcortical Structural Atlas [51] and merged to one bilateral amygdala seed. Afterwards, dual regression [52] was applied using the bilateral amygdala seed. This resulted in subject- and timepoint-specific whole-brain regression maps with respect to the bilateral amygdala.

### 2.9. Statistical Analysis

To Test for Baseline Differences between Groups, Participants’ Characteristics Were Analyzed Using an Independent *t*-Test Using SPSS 25 (SPSS Inc., Chicago, IL, USA).

#### 2.9.1. Physiological, Behavioral and Structural Data

For the analysis of physiological (relVO_2max_), behavioral (PANAS, STAI state and MoodMeter^®^), and structural MRI (amygdala nuclei) data, a linear mixed effects (LME) model was used (package lme4 [53]; software: RStudio (http://www.rstudio.com/; accessed on 2 April 2022). The covariates age and sex (as fixed effects) and a random intercept were added to account for random individual level effects. Additionally, the eTIV was added as a covariate to account for individual brain sizes for the analysis of the structural data. In the case of significant main effects (time, group or time by group interaction), an additional LME model with time as categorical variable was set up. This enabled us to calculate post hoc tests between timepoints within each group and between groups at each timepoint. Post hoc tests were performed using the emmeans package [54], and the multivariate t-distribution method was used to adjust *p*-values. The degrees-of-freedom method was Kenward-Roger [55]. Results of the post hoc tests were reported with Cohen’s *d* as an effect size [56].

#### 2.9.2. Resting-State Functional Connectivity

Whole-brain regression maps were inserted in an LME model using AFNI´s 3dLMEr toolbox (https://afni.nimh.nih.gov/pub/dist/doc/program_help/3dLMEr.html; accessed on 2 April 2022), investigating the longitudinal FC changes between the amygdala and the rest of the brain. The covariates age and sex (as fixed effects) and a random intercept were added to account for random individual level effects. The cluster level FWE method 3dClustSim (https://afni.nimh.nih.gov/pub/dist/doc/program_help/3dClustSim.html; accessed on 2 April 2022) was used to identify significant clusters of main effects and post hoc analyses. Clusters were considered significant at a voxel-level *p* < 0.001 (uncorrected) and at an alpha-level <0.05. Additionally, post hoc comparisons between timepoints within each group (paired *t*-test) and between groups (independent *t*-test) were performed and corrected for multiple comparisons (Bonferroni) using SPSS. Finally, for exploratory analysis, a more liberal threshold of *p* < 0.001 uncorrected with a cluster size (k) of k ≥ 10 voxels was set to search for trends.

#### 2.9.3. Correlation Analyses

Explorative correlation analyses between the change in FC of the amygdala, in relVO_2max_, and possible significant behavioral changes (PANAS, STAI state and/or MoodMeter^®^) were performed. Therefore, differences between T0 and T2, T0 and T4, and T0 and T6 were calculated by subtracting baseline values from the follow-up assessments. Due to the small sample sizes, we only correlated the values of both groups (IG and CG) together. Results of *p* < 0.05 (two-tailed) were considered significant.

## 3. Results

### 3.1. Participants

27 subjects in the IG and 15 subjects in the CG received the allocated intervention. During the course of the study, 14 subjects dropped out within the first four months or needed to be excluded from the final analyses. The reasons were loss of interest or not enough time (*N* = 9), elevated BDI in the course of the study (*N* = 2), injury due to private activities (*N* = 1), not possible to perform 3T measurement (*N* = 1), or not enough training counts due to illness (*N* = 1). This resulted in a final sample size of *N* = 28 subjects: *N* = 18 in the IG and *N* = 10 in the CG. Moreover, MRI scans could not be performed at T6 for two subjects due to newly appearing MRI contraindications (*N* = 1) and a dropout after T4 (*N* = 1), resulting in *N* = 26 subjects (IG: *N* = 16, CG: *N* = 10) for T6. Only one subject in the exercise group was a smoker (FTND value: 4, indicating a low level of dependence on nicotine). Participants’ characteristics are summarized in Table 1. The independent *t*-test showed no significant baseline differences between groups for the presented variables. 

### 3.2. Physiological Data—relVO_2max_

The IG performed, on average, 60.0 ± 11.1 (77 ± 14%) trainings out of 78 planned. Due to artifacts, relVO_2max_ values of two participants in the IG could not be determined. These participants were therefore excluded from the analysis of the relVO_2max_ data. 

**Figure 2:**
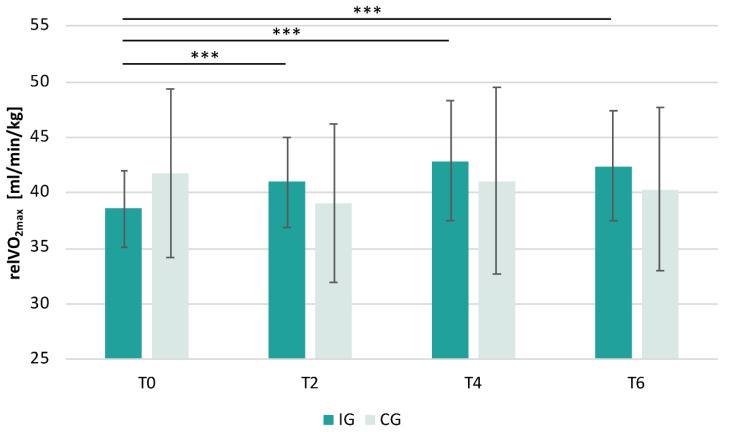
Change of relVO_2max_ over time in IG and CG. Mean ± standard deviation; number of subjects in the IG at T0: *N* = 16, T2: *N* = 16, T4: *N* = 16, T6: *N* = 15 and in the CG at T0: *N* = 10, T2: *N* = 7, T4: *N* = 9, T6: *N* = 10. *** *p* < 0.001.

Data showed an increase in relVO_2max_ in the IG and a slight decrease in the CG (Figure 2). The LME model for examining the effects of the exercise intervention on relVO_2max_ revealed a significant effect of time (F(1,71.05) = 11.91, *p* < 0.001) and time by group interaction (F(1,71.05) = 37.30, *p* < 0.001). However, there was no significant effect of group (F(1,25.04) = 0.22, *p* = 0.641). Moreover, a significant effect of sex was found (F(1,22.00) = 17.26, *p* < 0.001), indicating higher relVO_2max_ values in males than in females. Age had no significant impact on relVO_2max_ (F(1,22.00) = 0.01, *p* = 0.919).

Post hoc tests within the IG showed a significant increase in relVO_2max_ from T0 to T2 (t(67.0) = 4.06, *p* < 0.001, d = 1.44), T0 to T4 (t(67.0) = 7.26, *p* < 0.001, d = 2.57), and T0 to T6 (t(67.1) = 7.20, *p* < 0.001, d = 2.60). In the CG, no significant changes were detected (T0 to T2: t(67.4) = −1.07, *p* = 0.707, d = −0.55; T0 to T4: t(67.2) = −0.83, *p* = 0.840, d = −0.39; T0 to T6: t(67.0) = −1.92, *p* = 0.231, d = −0.86). Comparing IG with CG revealed no significant differences at T0 (t(26.5) = −0.78, *p* = 0.681, d = −0.90), T2 (t(28.6) = 0.93, *p* = 0.579, d = 1.08), T4 (t(27.1) = 1.78, *p* = 0.164, d = 2.05), and T6 (t(26.7) = 2.22, *p* = 0.072, d = 2.56).

### 3.3. Questionnaires

#### 3.3.1. STAI State

The STAI state showed a decrease in both groups from T0 to T6 (Table 2). The LME model for examining the effects of the exercise intervention on the STAI state revealed a significant effect of time (F(1,74.16) = 8.53, *p* = 0.005). However, there was no significant effect of group (F(1,33.65) = 0.07, *p* = 0.795) or time by group interaction (F(1,74.15) = 0.01, *p* = 0.936). A significant effect of sex was found (F(1,24.07) = 4.80, *p* = 0.038), indicating higher anxiety values in males than in females. Age had no impact on the STAI state (F(1,23.87) = 0.17, *p* = 0.682).

Post hoc pairwise comparisons revealed no significant effects in either of the groups (IG: T0 to T2 (t(70.4) = −0.67, *p* = 0.908, d = −0.24), T0 to T4 (t(70.0) = −1.02, *p* = 0.740, d = −0.34, T0 and T6 (t(70.2) = −2.62, *p* = 0.052, d = −0.89)); CG: T0 to T2 (t(71.0) = −2.60, *p* = 0.053, d = −1.32), T0 to T4 (t(70.6) = −0.81, *p* = 0.848, d = −0.38), T0 to T6 (t(70.0) = −2.40, *p* = 0.086, d = −1.08).

The PANAS PA showed slight increases over time (Table 2); however, no significant main effects were detected (time: F(1,74.25) = 1.15, *p* = 0.287; group: F(1,33.31) = 0.08, *p* = 0.776; time by group interaction: F(1,74.25) = 0.00, *p* = 0.951). Sex also showed no significant effect (F(1,24.17) = 3.49, *p* = 0.074). However, the variable age revealed a significant effect (F(1,23.97) = 7.97, *p* = 0.009), indicating that older individuals had higher PA scores than younger individuals. 

The PANAS NA score showed slight decreases over time in both groups (Table 2), resulting in a significant main effect of time (F(1,73.99) = 5.09, *p* = 0.027). No other significant main effects were found (group: F(1,52.08) = 0.97, *p* = 0.329; time by group interaction: F(1,73.97) = 1.34, *p* = 0.250, age: F(1,23.17) = 1.27, *p* = 0.272; sex: F(1,23.73) = 1.99, *p* = 0.171).

Post hoc tests revealed no significant effects in either of the groups when comparing the timepoints: T0 and T2 (IG: t(71.3) = −0.91, *p* = 0.801, d = −0.32; CG: t(72.7) = −1.78, *p* = 0.291, d = −0.90), T0 and T4 (IG: t(70.1) = 0.21, *p* = 0.997, d = 0.07; CG: t(71.4) = −2.09, *p* = 0.167, d = −0.97), and T0 and T6 (IG: t(70.5) = −1.34, *p* = 0.541, d = −0.45; CG: t(70.1) = −2.15, *p* = 0.148, d = −0.96). 

#### 3.3.2. MoodMeter^®^

MoodMeter^®^ statistics revealed a main effect of time for the subdimensions physical fitness and physical flexibility and an effect of age in physical fitness. Values of the MoodMeter^®^ dimensions are summarized in Table 2, and statistics of the main effects are summarized in Table 3. Post hoc tests are reported in the supplement. 

### 3.4. Structural MRI

The analysis of structural changes in the amygdala and its subfields did not reveal any exercise-induced changes. However, single main effects of age and group were found. Data and statistics are summarized in the supplement (Figures S1 and S2; Tables S1 and S2).

### 3.5. Functional Connectivity

**Figure 3:**
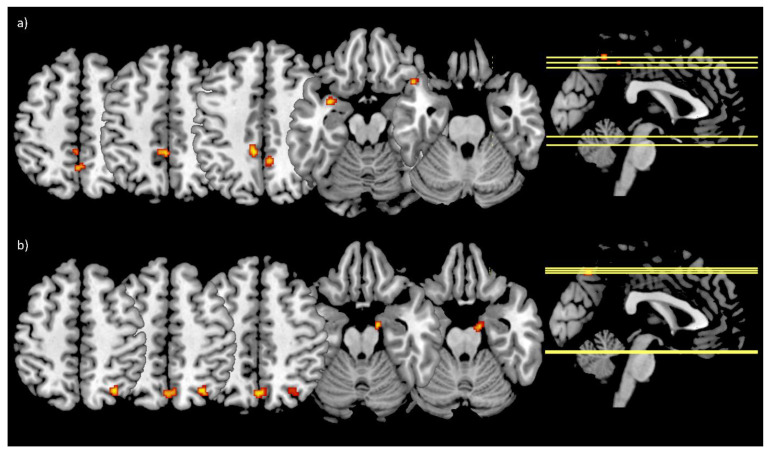
Main effects of the LME model for the bilateral amygdala FC changes. (**a**) Showing the clusters (red-yellow) derived from the time by group interaction within the left middle cingulate cortex, the precuneus, and the left temporal pole; (**b**) shows the clusters (red-yellow) from the main effect of time within the precuneus, the right superior parietal lobule, and the right parahippocampal gyrus; threshold: *p* < 0.001 uncorrected, k ≥ 10 voxels; IG: *N* = 18 (*N* = 16 at T6), CG: *N* = 10.

FC analyses revealed no significant main effects (time, group, or time by group interaction) for the bilateral amygdala seed. To search for trends the threshold exploratively was set to *p* < 0.001 uncorrected with a cluster size threshold of k ≥ 10 voxels. With the lower threshold, the time by group interaction showed an effect in the left middle cingulate cortex (peak voxel: [−8 −40 44]; k = 47), the precuneus (peak voxels: [6 −48 44], k = 16 and [0 −54 52], k = 13), and the left temporal pole (peak voxels: [−32 4 −16], k = 19 and [−48 20 −26], k = 12) (Figure 3a). Furthermore, the effect of time revealed an effect in the precuneus (peak voxel: [0 −68 50], k = 23), the right superior parietal lobule (peak voxel: [30 −64 52], k = 21), and the right parahippocampal gyrus (peak voxel: [14 −8 −20], k = 11) (Figure 3b). 

**Figure 4:**
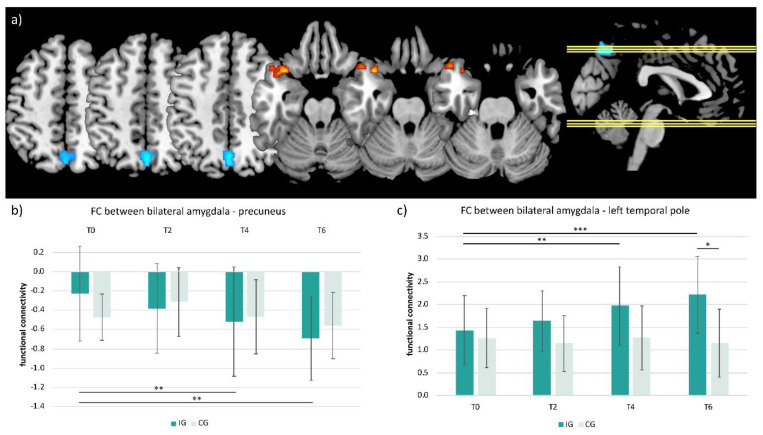
Significant bilateral amygdala FC changes derived from the time effect within the intervention group. (**a**) Showing the clusters within the precuneus (blue) and the temporal pole (red-yellow) (threshold: *p* < 0.001 uncorrected, alpha level = 0.05, k ≥ 58 voxels); (**b**) shows the amygdala–precuneus FC (mean ± standard deviation) extracted from the precuneus cluster for all timepoints (T0, T2, T4, and T6) and both groups (IG and CG); (**c**) shows the amygdala–right temporal pole FC (mean ± standard deviation) extracted from the cluster in the temporal pole for all timepoints (T0, T2, T4, and T6) and both groups (IG and CG; FC = resting state functional connectivity; T0, T2, T4, T6 = examination day after 0, 2, 4, 6 months; IG: *N* = 18 (*N* = 16 at T6), CG: *N* = 10; *** *p* < 0.001, ** *p* < 0.01, * *p* < 0.05.

Due to these uncorrected findings in the interaction, the 3dLMEr model was further explored. A significant main effect of time was found for the IG, showing an anticorrelation between the bilateral amygdala and the precuneus time course (peak voxel: [2 −66 46]; k = 102; F-statistics = −4.86; Figure 4a) and a positive correlation between the bilateral amygdala and the left temporal pole time course (peak voxel: [−34 16 −22]; k = 84; F-statistics = 5.24; Figure 4a) (threshold: *p* < 0.001, alpha level = 0.05; k ≥ 58 voxels). FC changes extracted from these clusters are presented in Figure 4b,c, showing step-wise changes of the amygdala-precuneus FC (i.e., stronger anticorrelation) in the IG as well as an increased positive FC (i.e., stronger positive correlation) between the bilateral amygdala and the left temporal pole after two, four, and six months, while no change was detected in the CG.

Post hoc tests showed that the FC changes over time between the bilateral amygdala and the precuneus cluster were significant from T0 to T4 and from T0 to T6 (*p* < 0.01) (Figure 4b). The same applies to the FC changes between the bilateral amygdala and the left temporal pole (T0 to T4: *p* < 0.01; T0 to T6: *p* < 0.001) (Figure 4c). Additionally, a significant difference between IG and CG was found at T6 (*p* < 0.05) (Figure 4c).

To explore if FC changes in significant clusters were driven by changes of the right or left amygdala FC, the analysis was repeated with separate seeds for the left and right amygdala. Analyses revealed that the FC changes of the bilateral amygdala were mainly driven by changes in the FC of the right amygdala (see supplement Figure S3, Tables S3 and S4).

### 3.6. Correlation Analyses

Explorative correlation analyses were performed for changes between the timepoints T0 and T4 and T0 and T6, as only after 4 months of exercise significant changes in FC occurred. Correlations were calculated between bilateral amygdala FC changes to the precuneus, bilateral amygdala FC changes to the temporal pole, relVO_2max_, and MoodMeter^®^ subdimensions, which showed a significant time effect (physical fitness, physical flexibility, and self-confidence). One outlier in the IG regarding relVO_2max_ was removed from the final analyses.

**Figure 5:**
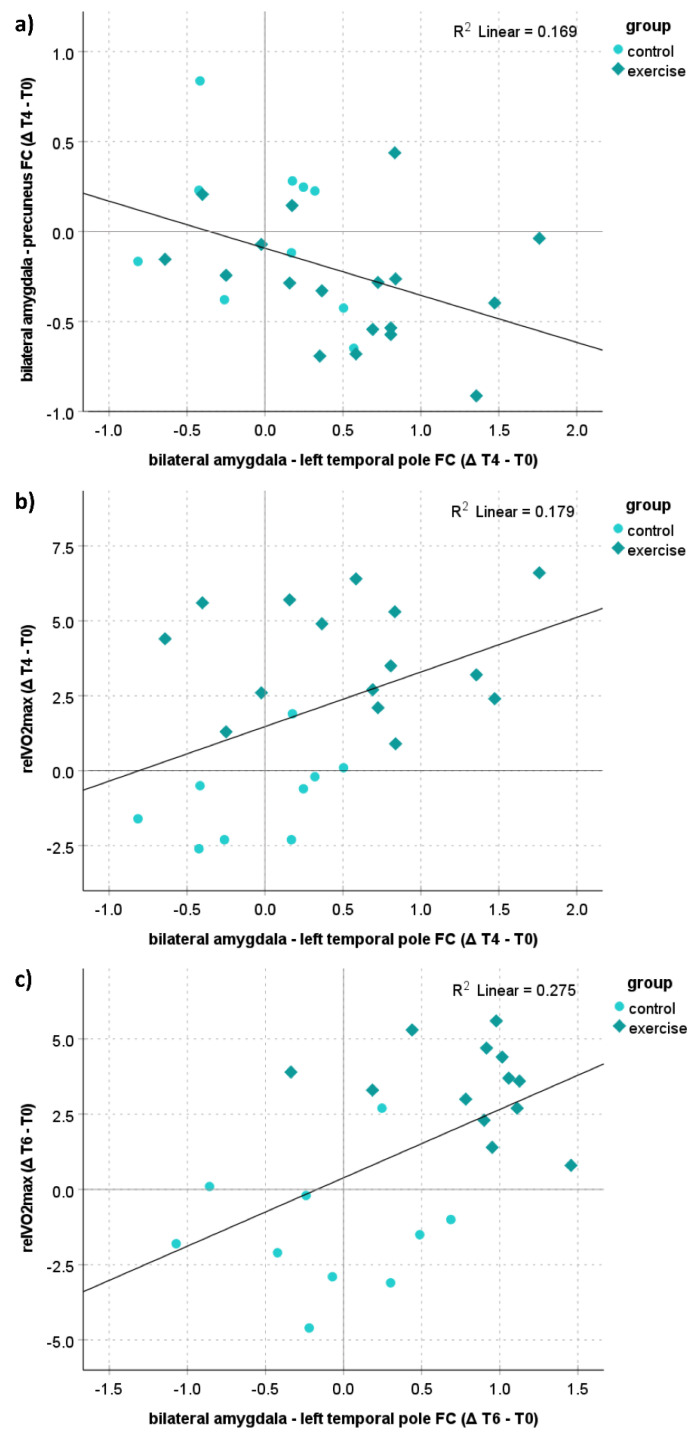
Correlations between physical fitness and FC. (**a**) Shows the correlation between the amygdala-precuneus FC and amygdala-temporal pole FC from T0 to T4; (**b**) shows the correlation between the relVO_2max_ [mL/min/kg] and amygdala-temporal pole FC from T0 to T4; (**c**) shows the correlation between the relVO_2max_ [mL/min/kg] and amygdala-temporal pole FC from T0 to T6.

Analyses over both groups together revealed a significant negative correlation between amygdala-precuneus FC and amygdala-temporal pole FC for the change from T0 to T4 (*p* = 0.030, r = −0.411, *N* = 28; Figure 5a) and a trend from T0 to T6 (*p* = 0.099, r = −0.331), indicating that a stronger anticorrelation between the amygdala and precuneus goes along with a stronger positive correlation between the amygdala and temporal pole. Moreover, a significant positive correlation between the changes of the amygdala-temporal pole FC and relVO_2max_ from T0 to T4 (*p* = 0.039, r = 0.423, *N* = 24; Figure 5b) and from T0 to T6 (*p* = 0.010, r = 0.524, *N* = 23; Figure 5c) were revealed, indicating that an increase in physical fitness induced by regular trainings goes along with increases in the amygdala-temporal pole FC. There were no significant correlations with any of the subdimensions of the MoodMeter^®^.

## 4. Discussion

As previous studies and network models of emotion converge on the eminent role of the amygdala, this study investigated the structural and functional changes of the amygdala induced by a 6-month exercise intervention in healthy untrained adults. Behavioral questionnaires assessing mood were examined at multiple timepoints and change-scores in various behavioral subdimensions were correlated with changes in MRI data. As expected, physical fitness improved exclusively in the IG, showing significant increases in relVO_2max_ at all timepoints compared to baseline, whereas no fitness change could be identified in the CG. RelVO_2max_ increased steadily in the IG until timepoint T4, before reaching a plateau. These objective fitness improvements were also partially mirrored in the subjective self-ratings of the MoodMeter^®^ for the sub-dimension physical fitness, where increases were revealed on post hoc testing in the IG between timepoints T0 and T6, along with increases in the sub-dimensions self-confidence (significant) and physical flexibility (trend only). Regarding affective measures, no significant differences between the two groups could be detected over time. Imaging data revealed no significant structural changes of the amygdala, including its sub-nuclei. However, at the functional level, the IG was characterized by significant FC changes of the bilateral amygdala after 4 and 6 months of extensive interval training: Data showed significant step-wise stronger amygdala-precuneus anticorrelation over time. Additionally, successive changes of the amygdala-temporal pole FC (stronger positive correlation) in the IG were observed. Effects in the FC were mainly driven by changes in the connection strength of the right amygdala. Importantly, the FC changes at T4 between the bilateral amygdala-temporal pole were significantly negatively correlated with the FC changes between the bilateral amygdala-precuneus, indicating inverse effects over time. At T6, this effect was only visible as a trend. Notably, changes in physical fitness (relVO_2max_) and in the bilateral amygdala-left temporal pole FC from the baseline to T4 and to T6 were significantly positively correlated, suggesting that observed findings were driven by exercise-induced changes in fitness. 

Despite the fact that this study did not reveal any significant behavioral effects regarding mood, informative FC-changes were encountered during and after 6 months of exercise. Specific modulation of amygdala FC with two anatomical regions known to show typical FC-changes in mood and anxiety disorders, namely the precuneus as part of the DMN and the temporal pole, were observed.

Converging evidence in the previous literature points towards increased amygdala-DMN rs-FC in patients with affective disorders: adolescents with depression show amygdala-precuneus hyperconnectivity [6]. For instance, repetitive negative ways of thinking such as rumination and worries are frequently encountered in depressive patients and go along with atypical functional hyperconnectivity between the DMN and subgenual PFC [57,58]. Hence, the stronger amygdala-precuneus anticorrelation found in this study could be interpreted as a beneficial stabilizing factor, mediating protective effects on affective networks via regular exercise.

The temporal pole is a distinct anatomical area involved in a variety of higher-order cognitive functions, including visual perception, autobiographical memory, language, face processing, and socioemotional functions [59,60]. Disruptions in these processes have been repeatedly associated with depression [61,62]. Moreover, the temporal pole is strongly connected to the amygdala [63], and structural and functional abnormalities of the temporal pole have also been found in patients with depression: Peng et al. found decreased GM in the temporal pole in MDD patients [64], and Tang et al. described a hypoconnectivity of the amygdala with the right inferior temporal gyrus (including the temporal pole) in depressed patients [6]. By demonstrating that exercise leads to increased FC between the amygdala and temporal pole, exercise could potentially counteract these characteristic FC changes in depression and might help to overcome the deficits described above.

The additional structural MRI analyses of the amygdala and its subfields did not reveal either volume increases or decreases. We have to point out that no other long-term exercise study we are aware of has so far reported significant structural plasticity of the amygdala. Rather, exercise-induced structural plasticity is well established for brain areas typically showing age-related deterioration and typically involved in cognitive performance [65,66]. In a recent meta-analysis in populations around 60 years, physical exercise was significantly associated with structural changes in the hippocampus/parahippocampus, while structural changes of the medial/superior prefrontal cortex were only found at trend levels [67]. Likewise, another metanalytical study on nine randomized controlled trials in healthy older adults showed significant regional volume increases in both the temporal (left superior temporal gyrus, left medial temporal gyrus) and the frontal (left IFG, right medial frontal gyrus, right and left superior frontal gyrus) regions, among others. Taken together, the findings indicate that structural plasticity induced by exercise is rather found in the temporal and frontal cortical areas than in the amygdala. It is, however, worth mentioning that a recent register study has suggested more widespread effects of exercise on whole-brain GM [68]. Hence, further research is needed to investigate gray matter volume changes beyond the hippocampus and the frontal cortex induced by exercise.

## 5. Limitations

One limitation is the rather small cohort of *N* = 28 subjects, and the especially small CG (*N* = 10). Further, given extensive maturation of key cortical areas in the investigated age range (18–35 years) and the mixture of sexes in a rather small cohort might have an effect on the data. Therefore, further research in bigger cohorts is warranted to confirm the exploratory results of this study. 

Opposite to Tozzi et al. [13], who found significant decreases in total mood disturbance after 4 months of exercise in the IG, we were not able to show significant interaction effects in the acquired mood questionnaires. One possible reason might be the floor effect in negative mood dimensions (STAI state and PANAS NA), as healthy cohorts typically do not show increased negative mood tendencies. Moreover, the mood questionnaires in this study are optimized for the measurement of situational mood states. Additional variance induced by moment-to-moment fluctuations may have reduced the chance of detecting more generalized mood changes in the participants. Regarding imaging findings, we have to acknowledge that, although the amygdala rs-FC changed significantly within the IG, no significant group by time interaction could be found at conservative statistical thresholds, possibly due to the small/imbalanced sample. Future longitudinal interventions studies are necessary in larger cohorts. 

## 6. Conclusions

The exercise-induced specific changes in the amygdala-FC to the precuneus and temporal pole suggest a shift of connectivity patterns into the opposite direction described in affective-disorder patient studies. The observed step-wise changes of the amygdala-precuneus FC and the successive changes of the amygdala-temporal pole FC appear as beneficial modulations of amygdala connectivity induced by repeated exercise. However, as these changes were observed in a population with relatively low pathological burden/symptoms, a transfer to patient studies is necessary to verify whether similar effects can be observed. The changes in relVO_2max_ and the amygdala-left temporal pole FC from the baseline to T4/T6 were significantly correlated, hence suggesting a modulation of amygdala-FC by fitness.

## Figures and Tables

**Table 1:** Descriptive characteristics at baseline.

Variable	Intervention *N* = 18 (m/f: 7/11)	Control *N* = 10 (m/f: 6/4)	*p*-Values Independent *t*-Test *
Age [years]	23.9 ± 3.9	23.7 ± 4.2	0.879
Height [cm]	173.6 ± 12.1	176.9 ± 7.9	0.447
Weight [kg]	69.9 ± 15.1	71.2 ± 14.1	0.816
BMI [kg/m^2^]	23.1 ± 3.7	22.7 ± 3.6	0.771
HR_max_ [bpm]	198.5 ± 7.6	200.8 ± 8.5	0.467
relVO_2max_ [mL/min/kg]	38.5 ± 3.4	41.7 ± 7.5	0.232
Education [years]	16.3 ± 3.1	15.8 ± 3.1	0.730
EHI [L.Q.]	74.2 ± 16.2	79.5 ± 13.3	0.390
BDI	2.6 ± 3.4	1.4 ± 1.5	0.224
STAI trait	33.9 ± 9.3	31.1 ± 5.8	0.390
WST IQ	107.0 ± 9.9	107.3 ± 8.8	0.937

Data presented as mean ± standard deviation. BDI = Beck Depression Inventory (0–10 points: no depression, 11–17 points: mild to moderate level of depressive symptoms, ≥18 clinically relevant); BMI = body mass index; HR_max_ = maximum heart rate in performance diagnostic; EHI = Edinburgh-Handedness-Inventory; WST IQ = verbal intelligence quotient of the German vocabulary test; L.Q. = Laterality Quotient (a value > +40 indicates right-handedness); relVO_2max_ = maximum oxygen consumption relative to body weight; STAI trait = trait anxiety of the State-Trait Anxiety Inventory (clinically relevant anxiety scores are ≥39); * two-sided.

**Table 2:** Behavioral data of the mood questionnaires. Mean ± standard deviation values for both groups (IG and CG) and for each timepoint (baseline (T0), after two (T2), four (T4), and six (T6) months).

Questionnaire	Dimension	Group	T0	T2	T4	T6
**STAI**	State anxiety	Intervention	33.3 ± 6.7	31.3 ± 5.9	32.1 ± 8.5	29.5 ± 5.4
		Control	35.9 ± 7.3	31.6 ± 3.3	34.7 ± 5.0	32.2 ± 5.2
**PANAS**	Positive affect scale	Intervention	28.4 ± 6.4	27.1 ± 7.1	26.5 ± 7.4	30.1 ± 8.5
		Control	25.4 ± 6.9	27.6 ± 7.4	25.9 ± 5.7	26.9 ± 7.0
	Negative affect scale	Intervention	11.6 ± 1.6	10.9 ± 1.1	11.7 ± 2.5	10.8 ± 1.3
		Control	12.7 ± 3.3	11.3 ± 1.5	11.2 ± 1.7	11.2 ± 2.1
**MoodMeter^®^**	**PEPS**	Intervention	3.4 ± 0.8	3.6 ± 0.5	3.6 ± 0.5	3.7 ± 0.6
		Control	3.1 ± 0.6	2.9 ± 0.4	3.1 ± 0.7	3.3 ± 0.7
	Physical energy	Intervention	3.9 ± 1.1	4.0 ± 0.9	3.9 ± 1.1	3.8 ± 1.1
		Control	4.0 ± 0.6	4.3 ± 0.7	4.0 ± 0.9	4.2 ± 0.7
	Physical fitness	Intervention	2.5 ± 1.1	2.8 ± 0.7	2.9 ± 0.9	3.0 ± 0.9
		Control	2.0 ± 1.0	1.8 ± 0.9	2.3 ± 0.8	2.3 ± 1.2
	Physical health	Intervention	4.4 ± 0.6	4.4 ± 0.7	4.3 ± 0.8	4.5 ± 0.6
		Control	4.0 ± 0.9	3.6 ± 1.0	3.8 ± 1.0	4.2 ± 1.0
	Physical flexibility	Intervention	2.9 ± 1.1	3.2 ± 0.7	3.2 ± 0.9	3.5 ± 0.7
		Control	2.3 ± 0.8	2.1 ± 1.0	2.4 ± 0.9	2.7 ± 0.9
	**PSYCHO**	Intervention	3.6 ± 1.1	3.8 ± 0.7	3.6 ± 1.0	3.7 ± 0.9
		Control	3.6 ± 0.5	3.7 ± 1.0	3.5 ± 0.7	3.6 ± 0.8
	Positive mood	Intervention	3.6 ± 1.2	3.8 ± 1.1	3.6 ± 1.3	3.8 ± 1.1
		Control	3.2 ± 1.2	3.5 ± 0.9	3.2 ± 1.1	3.4 ± 0.9
	Calmness	Intervention	4.0 ± 0.9	4.3 ± 0.6	4.2 ± 0.8	4.2 ± 0.9
		Control	3.7 ± 1.3	3.9 ± 0.9	3.6 ± 1.3	4.0 ± 1.0
	Recovery	Intervention	3.2 ± 1.4	3.3 ± 1.0	3.4 ± 1.2	3.4 ± 1.1
		Control	3.4 ± 0.8	3.3 ± 1.3	3.2 ± 0.9	3.1 ± 1.1
	Relaxation	Intervention	3.6 ± 1.2	3.6 ± 1.1	3.4 ± 1.3	3.4 ± 1.4
		Control	4.1 ± 0.8	4.1 ± 1.2	3.8 ± 1.0	4.1 ± 0.9
	**MOT**	Intervention	3.3 ± 0.9	3.4 ± 0.7	3.3 ± 0.8	3.5 ± 0.7
		Control	2.7 ± 0.9	2.7 ± 0.9	2.8 ± 0.6	2.9 ± 1.1
	Willingness to seek contact	Intervention	3.6 ± 0.8	3.4 ± 1.2	3.2 ± 0.9	3.4 ± 1.0
		Control	2.6 ± 1.3	2.7 ± 0.6	2.6 ± 0.6	2.7 ± 1.4
	Social acceptance	Intervention	3.5 ± 1.1	3.7 ± 0.8	3.5 ± 0.9	3.8 ± 0.9
		Control	3.1 ± 1.1	3.3 ± 1.3	3.1 ± 0.9	3.2 ± 1.5
	Readiness to strain	Intervention	2.9 ± 1.3	3.1 ± 0.9	3.0 ± 1.0	2.8 ± 1.1
		Control	2.4 ± 0.9	2.1 ± 1.2	2.6 ± 0.6	2.3 ± 1.0
	Self-confidence	Intervention	3.1 ± 0.9	3.4 ± 0.8	3.5 ± 0.7	3.9 ± 0.7
		Control	2.8 ± 0.9	2.6 ± 1.1	3.1 ± 1.1	3.6 ± 1.1

MOT = motivational state, NA = negative Affect, PANAS = Positive and Negative Affect Scale, PA = positive affect, PEPS = perceived physical state, PSYCHO = psychological strain, STAI = state-trait anxiety inventory.

**Table 3:** Statistics of the MoodMeter^®^ questionnaire.

Dimension	Effect of Time			Effect of Group			Time × Group Interaction			Effect of Sex			Effect of Age		
	df	F	*p*-Value	df	F	*p*-Value	df	F	*p*-Value	df	F	*p*-Value	df	F	*p*-Value
**Perceived physical state (PEPS)**	1, 73.18	3.66	0.060	1, 40.87	1.81	0.187	1, 73.17	0.03	0.868	1, 23.87	0.37	0.551	1, 23.53	1.57	0.223
Physical energy	1, 72.84	0.00	0.950	1, 46.15	0.36	0.550	1, 72.83	0.58	0.447	1, 23.43	1.34	0.260	1, 22.98	0.18	0.673
Physical fitness	1, 73.54	7.58	0.007 **	1, 37.81	2.20	0.146	1, 73.53	0.35	0.558	1, 24.30	0.06	0.808	1, 24.02	4.46	0.045 *
Physical health	1, 73.34	0.77	0.384	1, 36.13	2.59	0.116	1, 73.34	0.49	0.488	1, 24.13	0.02	0.896	1, 23.88	0.08	0.780
Physical flexibility	1, 73.56	6.40	0.014 *	1, 48.43	3.43	0.070	1, 73.55	0.17	0.685	1, 24.12	0.39	0.540	1, 23.64	2.73	0.112
**Psychological strain (PSYCHO)**	1, 73.30	0.00	0.951	1, 44.48	0.01	0.932	1, 73.29	0.00	0.953	1, 23.29	1.72	0.202	1, 23.52	1.74	0.200
Positive mood	1, 73.42	0.27	0.605	1, 38.31	0.26	0.610	1, 73.41	0.03	0.862	1, 24.17	4.05	0.056	1, 23.88	3.37	0.079
Calmness	1, 73.78	0.60	0.442	1, 47.31	1.22	0.276	1, 73.77	0.08	0.781	1, 24.38	0.69	0.416	1, 23.92	0.37	0.550
Recovery	1, 73.34	0.06	0.808	1, 52.48	0.35	0.558	1, 73.33	0.84	0.363	1, 23.82	0.99	0.330	1, 23.26	0.76	0.391
Relaxation	1, 73.15	0.50	0.484	1, 70.58	1.20	0.277	1, 73.13	0.31	0.581	1, 23.22	0.21	0.651	1, 22.30	1.11	0.304
**Motivational state** **(MOT)**	1, 73.29	3.10	0.083	1, 32.77	2.75	0.107	1, 73.28	0.19	0.668	1, 24.13	1.42	0.246	1, 23.95	1.35	0.257
Willingness to seek contact	1, 73.43	0.00	0.994	1, 43.31	4.84	0.033 *	1, 73.42	0.59	0.444	1, 24.09	3.89	0.060	1, 23.70	0.22	0.646
Social acceptance	1, 73.24	0.81	0.371	1, 31.77	0.52	0.476	1, 73.23	0.35	0.556	1, 24.10	1.26	0.273	1, 23.93	0.28	0.604
Readiness to strain	1, 73.42	0.11	0.744	1, 40.17	1.45	0.236	1, 73.42	0.12	0.732	1, 24.14	1.47	0.237	1, 23.81	4.88	0.037 *
Self-confidence	1, 73.34	31.19	<0.001 ***	1, 33.99	2.21	0.147	1, 73.33	0,33	0.570	1, 24.16	0.06	0.803	1, 23.95	0.78	0.385

df = degrees of freedom; F = F-value; * *p* < 0.05, ** *p* < 0.01, *** *p* < 0.001. Bold to highlight the statistically significant values in this large table.

## Data Availability

The data presented in this study are available upon request from the corresponding author. The data are not publicly available due to privacy and/or ethical restrictions.

## Supplementary Materials

The following supporting information can be downloaded at: https://www.mdpi.com/article/10.3390/ijerph19106078/s1, Results: MoodMeter^®^, Structural data, Functional connectivity of the right amygdala seed, Functional connectivity of the left amygdala seed; Figure S1: Volumes of bilateral amygdala over time for IG and CG; Figure S2: Volumes of left and right amygdala over time for IG and CG; Figure S3: Significant right amygdala FC changes derived from the time effect within the intervention group; Table S1: Statistics of the LME model for the bilateral amygdala volumes; Table S2: Statistics of the LME model for the left and right amygdala volumes; Table S3: Trends derived from the 3dLMEr model for the right amygdala seed region; Table S4: Trends derived from the 3dLMEr model for the left amygdala seed region.
