# Supplementary material for: Effects of a 6-Month Aerobic Exercise Intervention on Mood and Amygdala Functional Plasticity in Young Untrained Subjects
Source: Int J Environ Res Public Health. 2022 May 17;19(10):6078. doi: 10.3390/ijerph19106078 (PMC9140773; doi:10.3390/ijerph19106078)
Supplement: Supplementary file 1 [file ijerph-19-06078-s001.zip › ijerph-1702196-supplementary.pdf]

Supplement

“Effects of a 6-month aerobic exercise intervention on mood and amygdala functional plasticity in young untrained subjects”

Authors

Angelika Maurer*, Julian Klein*, Jannik Claus, Neeraj Upadhyay, Leonie Henschel, Jason A. Martin, Lukas Scheef, Marcel Daamen, Theresa Schörkmaier, Rüdiger Stirnberg, Tony Stöcker, Alexander Radbruch, Ulrike Attenberger, Martin Reuter, Henning Boecker

MoodMeter®

The statistics of the dimension PEPS did not show any significant main effects, although it showed a strong trend for the main effect of time. However, the ANOVA for the sub-dimension physical fitness revealed a significant effect of time. Post hoc testing revealed no significant effect in the IG between timepoints T0 vs T2 ($t(69.8) = 1.81$, $p = 0.278$, $d = 0.65$), but showed a strong trend between timepoints T0 vs T4 ($t(69.2) = 2.57$, $p = 0.057$, $d = 0.87$) and a significant increase in the sub-dimension physical fitness between timepoints T0 vs T6 ($t(69.5) = 2.65$, $p = 0.048$, $d = 0.92$). In the CG no significant effects between the four timepoints were detected (T0 vs T2: $t(70.4) = -0.55$, $p = 0.947$, $d = -0.28$; T0 vs T4: $t(69.8) = 1.44$, $p = 0.476$, $d = 0.67$; T0 vs T6: $t(69.0) = 0.88$, $p = 0.816$, $d = 0.39$). Moreover, a significant main effect of age was detected.

A significant effect of time was also detected in the sub-dimension physical flexibility. Post hoc testing in the IG revealed a trend (increase) between the timepoints T0 and T6 ($t(69.9) = 2.50$, $p = 0.068$, $d = 0.86$), while no effects were seen between T0 vs T2 ($t(70.5) = 1.40$, $p = 0.505$, $d = 0.50$) and T0 vs T4 ($t(69.5) = 1.42$, $p = 0.494$, $d = 0.48$). In the CG no significant effects between the four timepoints were detected (T0 vs T2: $t(71.5) = 0.02$, $p = 1.000$, $d = 0.01$; T0 vs T4: $t(70.3) = 0.49$, $p = 0.961$, $d = 0.23$; T0 vs T6: $t(69.1) = 1.25$, $p = 0.594$, $d = 0.56$).

The sub-dimensions physical energy and physical health did not reveal any significant changes. The dimension MOT also did not show any significant effects, although a trend was found for the main effect of time. However, the sub-dimension willingness to seek contact revealed a significant main effect of group, showing higher values in the IG than in the CG. Post hoc testing revealed no significant effects between the groups at any timepoint (T0: $t(54.0) = 2.04$, $p = 0.147$, $d = 1.14$; T2: $t(66.7) = 1.77$, $p = 0.245$, $d = 1.09$; T4: $t(56.0) = 1.42$, $p = 0.437$, $d = 0.80$; T6: $t(54.3) = 1.38$, $p = 0.458$, $d = 0.77$).

A significant main effect of time was detected in the sub-dimension self-confidence. Post hoc testing in the IG revealed a significant increase only between the timepoints T0 and T6 ($t(69.3) = 4.10$, $p < 0.001$, $d = 1.42$), while no effects were seen between T0 vs T2 ($t(69.6) = 0.92$, $p = 0.797$, $d = 0.33$) and T0 vs T4 ($t(69.2) = 1.88$, $p = 0.248$, $d = 0.64$). In the CG no significant effects were found between T0 vs T2 ($t(70.0) = -0.57$, $p = 0.940$, $d = -0.29$) and T0 vs T4 ($t(69.6) = 1.41$, $p = 0.498$, $d = 0.65$), but a significant increase was observed between T0 vs T6 ($t(69.0) = 3.58$, $p = 0.004$, $d = 1.60$).

The sub-dimension readiness to strain only revealed a main effect of age. The sub-dimension social acceptance revealed no significant changes.

The dimension PSYCHO and its sub-dimensions (positive mood, calmness, recovery, relaxation) did not reveal any significant changes.

Structural data

Volumes of the bilateral amygdala are shown in **Figure S1** and for left and right amygdala separately in **Figure S2**. Analysis of structural changes in the amygdala induced by exercise revealed no significant main effect of time, group, sex or time by group interaction. This was the case for the bilateral amygdala volume, as well as separated volumes for left and right amygdala. However, a strong trend for the interaction group x time has been detected for the left accessory basal nucleus ($p = 0.089$) (**Table S2**).

A significant main effect of group has been detected for the bilateral central nucleus (**Table S1**). Moreover, significant main effects of age have been found for bilateral amygdala nuclei, namely the paralaminar nucleus, the basal nucleus and the whole amygdala (**Table S1**). For separated left and right amygdala nuclei significant effects of age have been observe for the left and right paralaminar nucleus, the left and right basal nucleus as well as the left whole amygdala (**Table S2**).

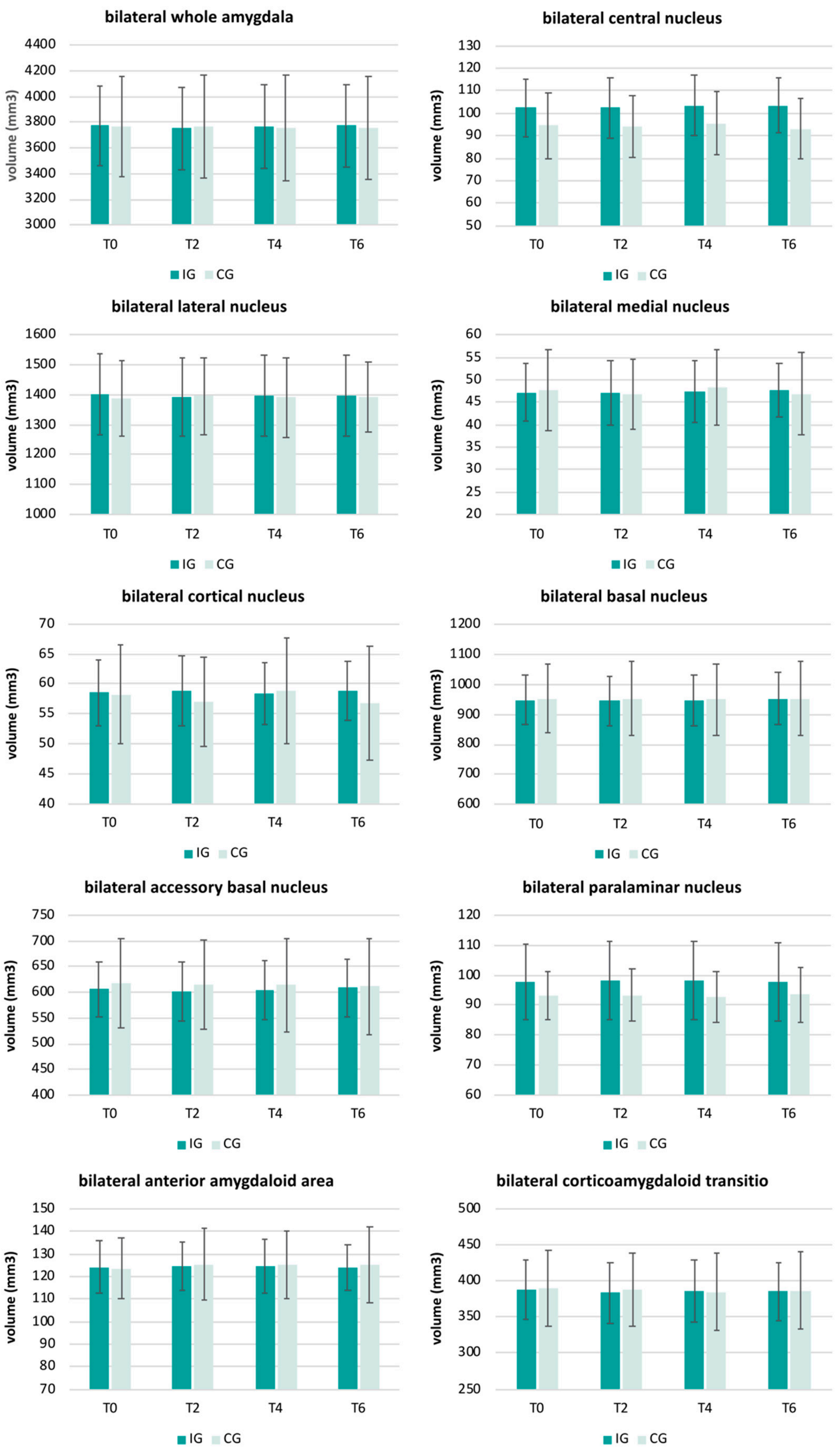

Figure S1: Volumes of bilateral amygdala over time for IG and CG. Data presented as mean ± standard deviation.

Table S1: Statistics of the LME model for the bilateral amygdala volumes.

bilateral regions	effect of time			effect of group			time x group interaction			effect of sex			effect of age			effect of eTIV		
	**df**	**F**	**p-value**	**df**	**F**	**p-value**	**df**	**F**	**p-value**	**df**	**F**	**p-value**	**df**	**F**	**p-value**			**p-value**
**whole Amygdala**	1,80.01	0.01	0.926	1,23.40	0.19	0.669	1,80.01	0.53	0.470	1,23.00	3.20	0.087	1,23.00	4.30	**0.049***	1,23.00	3.55	0.072
**central nucleus**	1,80.12	0.00	0.957	1,27.20	5.00	**0.034***	1,80.12	0.66	0.418	1,22.05	2.41	0.134	1,23.02	0.23	0.633	1,23.03	9.35	**0.006****
**lateral nucleus**	1,80.01	0.11	0.743	1,23.51	0.33	0.572	1,80.01	0.04	0.846	1,23.00	2.53	0.125	1,23.00	2.49	0.129	1,23.00	1.64	0.214
**medial nucleus**	1,80.09	0.03	0.869	1,29.42	0.00	0.961	1,80.09	0.47	0.497	1,22.99	1.22	0.280	1,22.94	0.50	0.488	1,22.96	5.45	**0.029***
**cortical nucleus**	1,80.09	0.29	0.594	1,26.06	0.42	0.521	1,80.09	0.66	0.420	1,23.04	3.92	0.060	1,23.02	0.15	0.701	1,23.03	2.24	0.148
**basal nucleus**	1,80.01	0.03	0.866	1,23.43	0.05	0.831	1,80.01	0.29	0.593	1,23.01	2.42	0.133	1,23.00	7.01	**0.014***	1,23.00	4.69	**0.041***
**accessory basal nucleus**	1,80.02	0.42	0.517	1,23.56	0.04	0.848	1,80.02	1.71	0.195	1,23.01	1.79	0.195	1.23.00	2.16	0.156	1,23.01	1.47	0.238
**paralaminar nucleus**	1,80.02	0.01	0.925	1,23.56	3.36	0.080	1,80.02	0.34	0.560	1,23.01	1.80	0.193	1,23.00	10.03	**0.004****	1,23.00	8.05	**0.009****
**anterior amygdaloid area**	1,80.02	0.91	0.344	1,23.92	0.05	0.820	1,80.02	0.43	0.515	1,23.01	0.65	0.430	1,23.00	1.42	0.246	1,23.00	0.53	0.474
**corticoamygdaloid transitio**	1,80.01	1.03	0.313	1,23.50	0.03	0.870	1,80.01	1.34	0.249	1,23.00	2.26	0.147	1,23.00	1.70	0.205	1,23.00	1.87	0.185

df = degrees of freedom; F = F-value; *p < 0.05, **p < 0.01

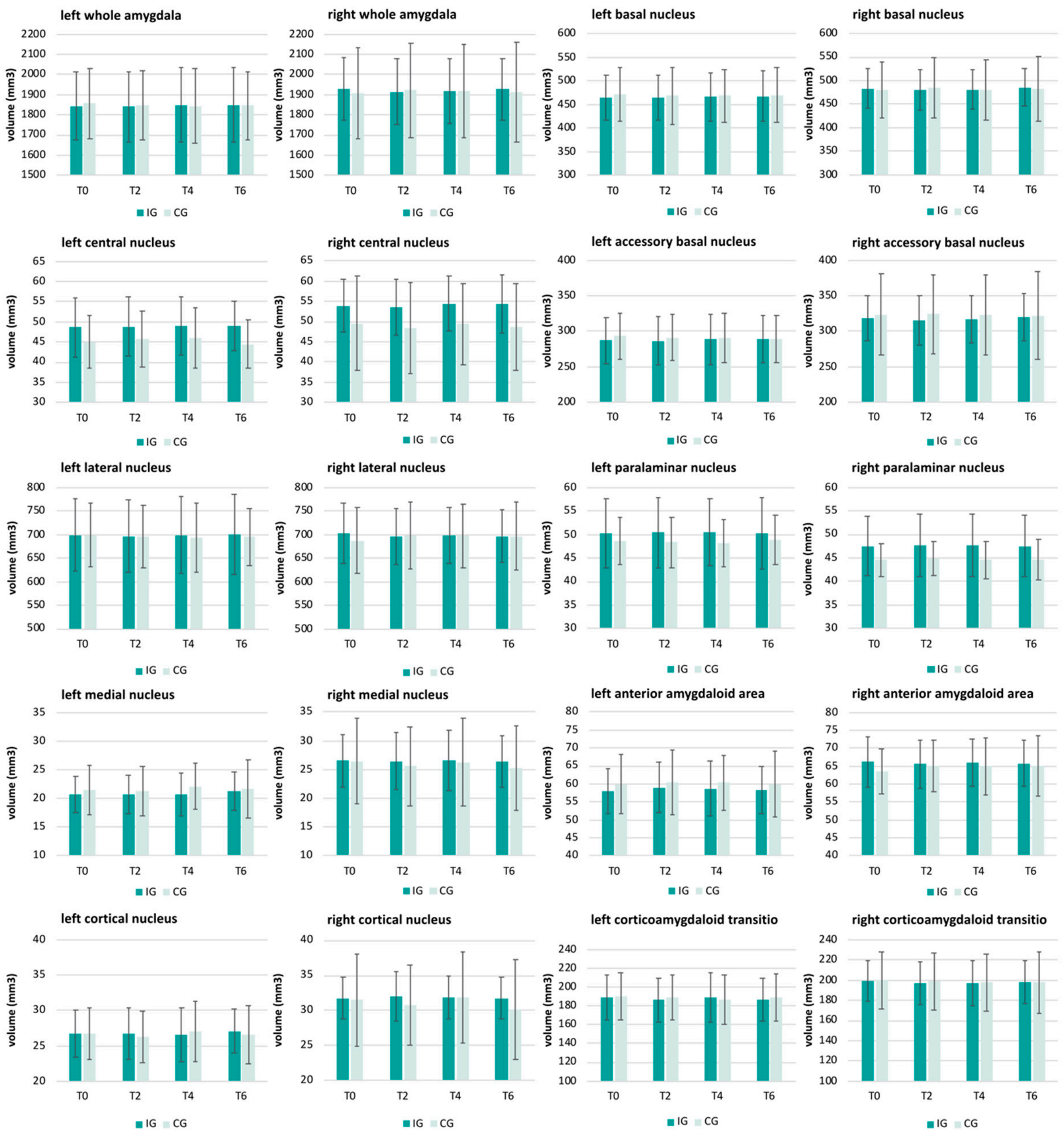

Figure S2: Volumes of left and right amygdala over time for IG and CG. Data presented as mean ± standard deviation.

Table S2: Statistics of the LME model for the left and right amygdala volumes.

Region	effect of time			effect of group			time x group interaction			effect of sex			effect of age			effect of eTIV		
	df	F	p-value	df	F	p-value	df	F	p-value	df	F	p-value	df	F	p-value	df	F	p-value
**L whole Amygdala**	1,80.02	0.00	0.945	1,23.75	0.01	0.904	1,80.02	2.24	0.139	1,23.01	1.82	0.190	1,23.00	5.79	**0.025***	1,23.00	4.54	**0.044***
**L central nucleus**	1,80.15	0.01	0.925	1,28.85	2.48	0.126	1,80.15	0.24	0.629	1,23.06	0.62	0.417	1,23.01	0.68	0.417	1,23.03	8.98	**0.006****
**L lateral nucleus**	1,80.02	0.06	0.803	1,23.68	0.08	0.782	1,80.02	1.60	0.209	1,23.01	1.48	0.236	1,23.00	3.09	0.092	1.23.00	1.47	0.238
**L medial nucleus**	1,80.09	1.10	0.298	1,30.27	0.52	0.476	1,80.09	0.05	0.816	1,22.98	0.36	0.554	1,22.93	0.38	0.544	1,22.95	1.91	0.181
**L cortical nucleus**	1,80.09	0.28	0.599	1,26.12	0.09	0.771	1,80.09	0.08	0.773	1,23.04	1.47	0.238	1,23.01	0.03	0.858	1,23.02	1.21	0.282
**L basal nucleus**	1,80.02	0.10	0.755	1,23.73	0.01	0.935	1,80.02	0.92	0.341	1,23.01	1.10	0.305	1,23.00	6.38	**0.019***	1,23.01	5.26	**0.031***
**L accessory basal nucleus**	1,80.02	0.19	0.667	1,23.76	0.10	0.756	1,80.02	2.97	0.089	1,23.01	0.50	0.485	1.23.00	2.02	0.169	1,23.01	1.50	0.233
**L paralaminar nucleus**	1,80.02	0.01	0.907	1,23.84	1.88	0.183	1,80.02	0.19	0.667	1,23.01	2.02	0.168	1,23.00	11.07	**0.003****	1,23.01	6.04	**0.022***
**L anterior amygdaloid area**	1,80.03	0.16	0.694	1,24.24	0.30	0.591	1,80.03	0.35	0.559	1,23.01	0.07	0.790	1,23.00	1.02	0.324	1,23.00	1.28	0.269
**L corticoamygdaloid transitio**	1,80.01	0.28	0.598	1,23.65	0.01	0.929	1,80.01	0.72	0.399	1,23.00	1.22	0.281	1,23.00	2.54	0.125	1,22.99	2.62	0.119
**R whole Amygdala**	1,80.01	0.01	0.938	1,23.47	0.44	0.515	1,80.01	0.26	0.614	1,23.01	3.94	0.059	1,23.00	2.40	0.135	1,23.00	2.13	0.158
**R central nucleus**	1,80.09	0.00	0.995	1,26.04	3.44	0.075	1,80.09	0.62	0.433	1,23.04	2.30	0.143	1.23.02	0.00	0.969	1,23.03	3.63	0.069
**R lateral nucleus**	1,80.02	0.51	0.477	1,24.06	0.71	0.407	1,80.02	2.32	0.132	1,23.01	3.31	0.082	1,23.00	1.28	0.270	1,23.00	1.39	0.251
**R medial nucleus**	1,80.06	0.68	0.412	1,26.51	0.24	0.629	1,80.06	0.80	0.375	1,23.01	3.30	0.082	1,22.98	0.17	0.680	1,22.99	3.49	0.075
**R cortical nucleus**	1,80.07	1.69	0.197	1,25.39	0.41	0.530	1,80.07	0.91	0.342	1,23.03	2.77	0.109	1,23.01	0.14	0.712	1,23.02	1.23	0.280
**R basal nucleus**	1,80.02	0.01	0.931	1,23.67	0.24	0.632	1,80.02	0.06	0.814	1,23.01	3.26	0.084	1,23.00	5.32	**0.030***	1,23.01	2.72	0.113
**R accessory basal nucleus**	1,80.02	0.40	0.529	1,23.53	0.00	0.959	1,80.02	0.20	0.657	1,23.01	2.30	0.143	1,23.00	1.35	0.258	1,23.00	0.83	0.370
**R paralaminar nucleus**	1,80.02	0.07	0.794	1,23.85	3.41	0.077	1,80.02	0.16	0.688	1,23.01	0.83	0.371	1,23.00	4.81	**0.039***	1,23.00	6.35	**0.019***
**R anterior amygdaloid area**	1,80.05	0.82	0.367	1,24.73	1.01	0.324	1,80.05	2.01	0.160	1,23.02	1.39	0.251	1.23.01	1.17	0.290	1,23.01	0.01	0.918
**R corticoamygdaloid transitio**	1,80.02	1.38	0.244	1,23.59	0.05	0.832	1,80.02	1.17	0.283	1,23.01	2.78	0.109	1,23.00	0.68	0.419	1,23.00	0.84	0.369

df = degrees of freedom; F = F-value; L = left: R = right; *p < 0.05, **p < 0.01

Functional connectivity of the right amygdala seed

Analysis using the 3dLMEr model revealed no significant main effects (time, group or time by group interaction) for the left or right amygdala seed. Due to the small sample size the threshold exploratively was set to $p < 0.001$ uncorrected with a cluster threshold of $k \geq 10$ voxels to search for trends.

In the model for the right amygdala seed, a trend in the time by group interaction was detected between the right amygdala and the middle cingulate gyrus, the precuneus and the left temporal pole (**Table S1**). Furthermore, the main effect of time also revealed a trend between the right amygdala and the left inferior frontal gyrus, left putamen, left calcarine gyrus, left hippocampus, right putamen, right postcentral gyrus, right precuneus, right superior temporal gyrus and right angular gyrus (**Table S1**). The main effect of group showed a trend between the right amygdala and the middle cingulate cortex (**Table S1**).

Due to these trends in the interaction, the 3dLMEr model was further explored. A significant main effect of time was found for the IG, showing an anticorrelation between the right amygdala and the precuneus (peak voxel: [2 −66 46]; $k = 161$; F-statistics = −4.92; **Figure S1a**) and a positive correlation between the right amygdala and the left temporal pole (peak voxel: [−34 16 −22]; $k = 102$; F-statistics = 5.48; **Figure S1a**) (threshold: $p < 0.001$, alpha level = 0.05; $k \geq 57$ voxels). Average β-values extracted from these clusters are shown in **Figure S1b and c**.

Post hoc tests showed that the FC change for the intervention over time effects between the right amygdala and the precuneus cluster was significant from T0 to T4 and from T0 to T6 ($p < 0.01$) (**Figure S1b**). The same applies to the FC changes between the right amygdala and the left temporal pole (T0 to T4: $p < 0.05$; T0 to T6: $p < 0.001$) (**Figure S1c**). Additionally, a significant difference between IG and CG was found at T6 ($p < 0.05$) (**Figure S1c**).

The main effect of time within the CG revealed a significant cluster in the right calcarine gyrus, extending into the lingual gyrus ($k = 71$ voxel) (threshold: $p < 0.001$, alpha level = 0.05; $k \geq 57$ voxels).

Table S3: Trends derived from the 3dLMEr model for the right amygdala seed region.

			Peak coordinates		
**Region**	**side**	**k**	**x**	**y**	**z**
**Interaction group x time**					
Middle cingulate cortex	L	48	−8	−42	44
Precuneus	L	29	−2	−60	50
Temporal pole	L	22	−32	4	−16
Precuneus	R	16	6	−48	44
**Main effect of Time**					
Inferior frontal gyrus (p. Orbitalis)	L	36	−34	18	−20
Putamen	L	14	−20	12	10
Calcarine gyrus	L	13	6	−90	0
Hippocampus	L	13	−26	−36	8
Putamen	R	13	26	10	12
Postcentral gyrus	R	13	62	−12	18
Precuneus	R	13	6	−66	50
Superior temporal gyrus	R	12	68	−16	8
Angular gyrus	R	11	30	−66	50
**Main effect of Group**					
Middle cingulate cortex	R	19	24	−36	36

L = left, R = right.

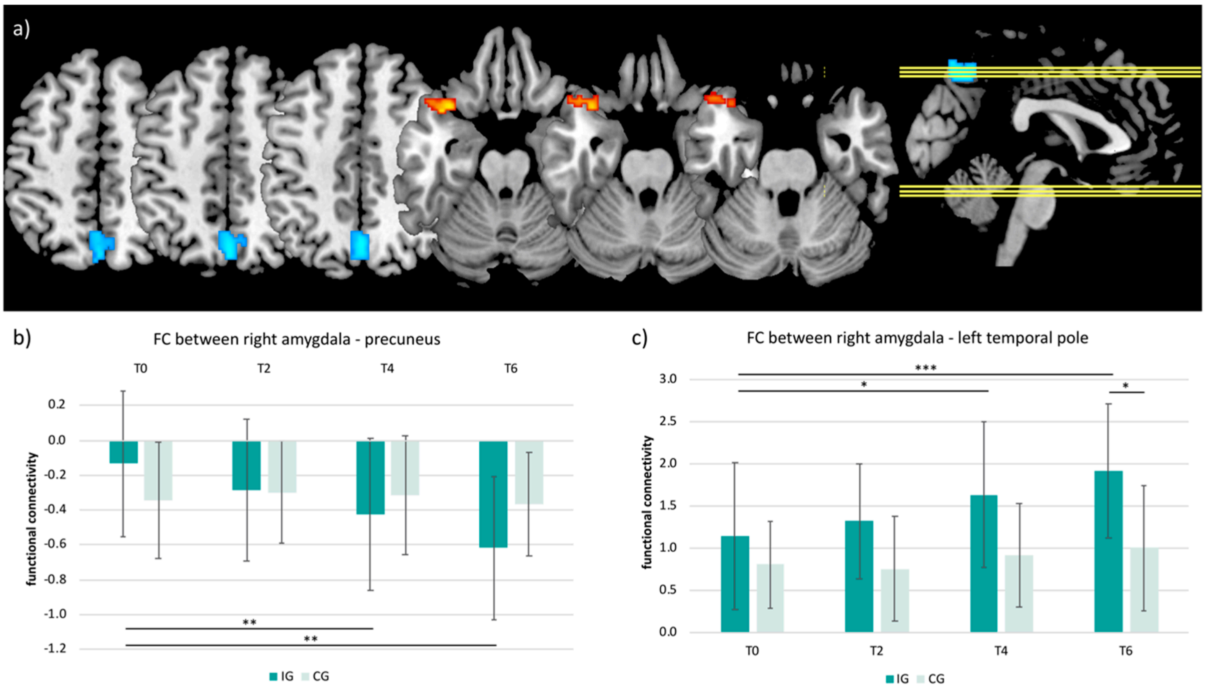

Figure S3: **Significant right amygdala FC changes derived from the time effect within the intervention group.** a) Showing the clusters within the Precuneus and the temporal pole (threshold: $p < 0.001$ uncorrected, alpha level = 0.05, $k \geq 57$ voxels); b) shows the ß-values (mean ± standard deviation) extracted from the precuneus cluster for all timepoints (T0, T2, T4 and T6) and both groups (IG and CG) representing the right amygdala – precuneus FC; c) shows the ß-values (mean ± standard deviation) extracted from the cluster in the temporal pole for all timepoints (T0, T2, T4 and T6) and both groups (IG and CG) representing the right amygdala – temporal pole FC; FC = resting-state functional connectivity; T0, T2, T4, T6 = Examination day after 0, 2, 4, 6 months; IG: N = 18 (N = 16 at T6), CG: N = 10.

Functional connectivity of the left amygdala seed

In the 3dLMEr model for the left amygdala seed, a trend in the time by group interaction was detected between the left amygdala and the left temporal pole, left and right superior orbital gyrus, right superior occipital gyrus, middle cingulate cortex, left inferior temporal gyrus, SMA, left superior frontal gyrus, and left middle orbital gyrus (**Table S2**). Furthermore, the main effect of time also revealed a trend between the left amygdala and the left insula, the left middle frontal gyrus, right parahippocampal gyrus, right middle occipital gyrus, and right middle frontal gyrus (**Table S2**). In the main effect of group, a trend was detected between the left amygdala and the left supramarginal gyrus (**Table S2**).

Post hoc analysis (main effect of time within the IG or CG) did not reveal any significant changes in left amygdala FC.

Table S4: Trends derived from the 3dLMEr model for the left amygdala seed region.

Region	side	k	Peak coordinates x	y	z
**<u>Interaction group x time</u>**					
Temporal pole	L	43	−48	18	−20
Superior orbital gyrus	R	24	16	30	−10
Superior occipital gyrus	R	23	24	−86	30
Superior orbital gyrus	L	18	−14	44	−12
Middle cingulate gyrus	L	18	−2	−40	50
Inferior temporal gyrus	L	17	−48	−36	−22
SMA	R	17	6	2	62
Superior frontal gyrus	L	15	−18	40	36
Middle orbital gyrus	L	14	−26	42	−20
Temporal pole	L	11	−32	2	−16
Middle cingulate cortex	R	11	18	−46	32
**<u>Main effect of Time</u>**					
Insula	L	26	−24	28	6
Middle frontal gyrus	L	14	−30	8	46
Parahippocampal gyrus	R	10	12	−10	−22
Middle occipital gyrus	R	10	40	−66	26
Middle frontal gyrus	R	10	36	26	48
**<u>Main effect of Group</u>**					
Supramarginal gyrus	L	11	−42	−48	28

L = left, R = right.
